# RYGB surgery has modest effects on intestinal morphology and gut hormone populations in the bypassed biliopancreatic limb but causes reciprocal changes in GLP-2 and PYY in the alimentary limb

**DOI:** 10.1371/journal.pone.0286062

**Published:** 2023-05-25

**Authors:** Ananyaa Sridhar, Dawood Khan, Jessie A. Elliott, Violetta Naughton, Peter R. Flatt, Nigel Irwin, Charlotte R. Moffett

**Affiliations:** 1 Biomedical Sciences Research Institute, School of Biomedical Sciences, Ulster University, Coleraine, N. Ireland, United Kingdom; 2 Department of Surgery, Trinity Centre for Health Sciences and St. James’s Hospital, Dublin, Ireland; Institute of Metabolic Science, University of Cambridge, UNITED KINGDOM

## Abstract

Roux-en-Y gastric-bypass (RYGB) induced alterations in intestinal morphology and gut-cell hormone expression profile in the bypassed biliopancreatic-limb (BPL) versus the alimentary-limbs (AL) are poorly characterised. This pilot study has therefore explored effects following RYGB in high-fat-diet (HFD) and normal-diet (ND) rats. Female Wistar rats (4-week-old) were fed HFD or ND for 23-weeks prior to RYGB or sham surgeries. Immunohistochemical analysis of excised tissue was conducted three-weeks post-surgery. After RYGB, intestinal morphology of the BPL in both HFD and ND groups was unchanged with exception of a small decrease in villi width in the ND-RYGB and crypt depth in the HFD-RYGB group. However, in the AL, villi width was decreased in ND-RYGB rats but increased in the HFD-RYGB group. In addition, crypt depth decreased after RYGB in the AL of HFD rats. GIP positive cells in either limb of both groups of rats were unchanged by RYGB. Similarly, there was little change in GLP-1 positive cells, apart from a small decrease of numbers in the villi of the BPL in HFD rats. RYGB increased GLP-2 cell numbers in the AL of ND-RYGB rats, including in both crypts and villi. This was associated with decreased numbers of cells expressing PYY in the AL of ND-RYGB rats. The BPL appears to maintain normal morphology and unchanged enteroendocrine cell populations despite being bypassed in RYGB-surgery. In contrast, in the AL, villi area is generally enhanced post-RYGB in ND rats with increased numbers of GLP-2 positive cells and decreased expression of PYY.

## Introduction

**Fig 1:**
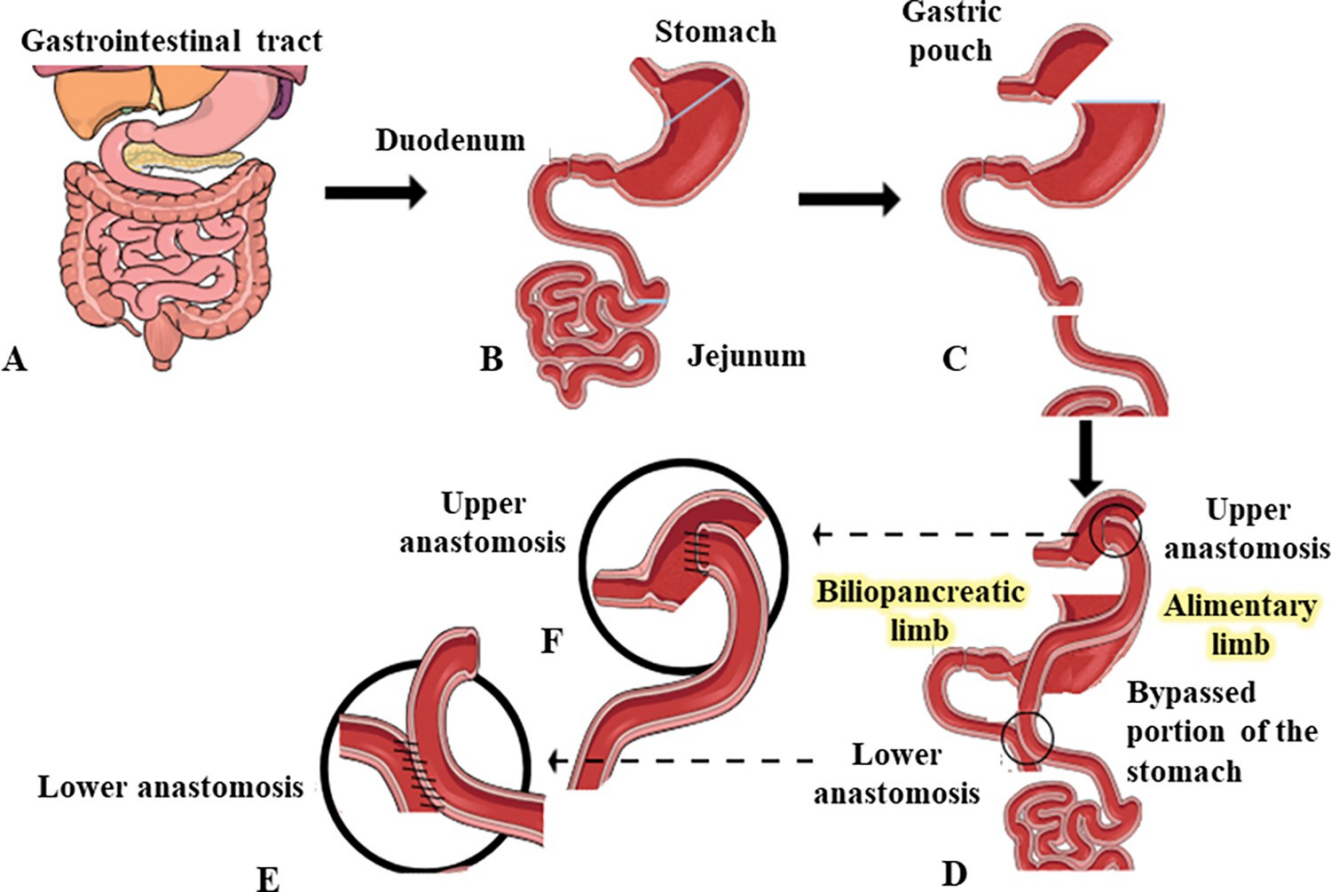
Diagrammatic representation of Roux-en-Y Gastric Bypass (RYGB) surgery procedure. (A) Normal pre-procedure gastrointestinal anatomy. (B,C) Identify duodenum or proximal jejunum passing under the colon and cut 10 cm from the duodenojejunal flexure to form the BPL. Identify cecum and follow ileum for approximately 30 cm. The BPL is ligated to the CC with retention stitching representing lower anastomosis (E). (C,D) The stomach is divided creating a smaller gastric pouch and the AL is ligated to the gastric pouch, representing the upper anastomosis (F).

Obesity is associated with various pathophysiological conditions such as diabetes mellitus, insulin resistance, atherosclerosis, hypertension and dyslipidemia [1]. Roux-en-Y Gastric Bypass (RYGB) surgery has emerged as a safe and successful bariatric procedure for morbid obesity [2]. Alleviation of insulin resistance, improved beta-cell function and substantial weight loss after RYGB improves blood glucose control often causing remission of type 2 diabetes [2]. The post-RYGB weight loss results from reduction in size of the stomach, re-direction of digesta passage and concomitant changes to digestion and absorption of ingesta via a surgically created alimentary limb (jejunal part of the small intestine; Fig 1). The stomach remnant together with duodenum, called the biliopancreatic limb (BPL) does not receive ingesta [3], however it is able to carry bile acids and pancreatic juice in joining the alimentary limb (AL) to form the common channel (CC). Although the above-described alteration of intestinal arrangement significantly reduces food intake contributing to weight loss [4], the key role of endocrine and related metabolic changes for diabetes remission after RYGB surgery has become more evident in recent times [5]. Indeed, substantial improvements of glucose control occur even before significant body weight reduction [5].

This phenomenon is further supported by two basic non-mutually exclusive theories. The first implicates loss of circulating factor(s) promoting insulin resistance resultant from the exclusion of the BPL [6]. The second postulates that rapid delivery of nutrients and bile to the distal AL results in increased secretion of restorative factor(s) [6]. Consistent with this, changes in hormone expression and secretion have been extensively documented showing decreased levels of circulating proximal gut hormones, such as glucose-dependent insulinotropic polypeptide (GIP) and enhanced concentrations of distal gut hormone secretions, including glucagon-like peptide-1 (GLP-1) [7,8]. These hormones exhibit a wide range of antidiabetic actions [9], but interestingly, ablation of GIP action has been linked to amelioration of spontaneous and diet-induced obesity in rodents [10]. Furthermore, other hormones secreted from the same L-cells as GLP-1, namely glucagon-like peptide-2 (GLP-2) and Peptide Tyrosine Tyrosine (PYY) have also been reported to circulate at increased concentrations after RYGB [8,11]. GLP-2 enhances integrity of the intestine and PYY incudes satiety, suggesting that these hormones may also contribute to the overall benefits of surgery. However, there is a scarcity of information regarding whether such RYGB induced effects directly correspond to alterations of the morphology and gut hormone cell population within both the BPL and AL. We employed female rats for the current investigations to ensure lack of sex-dependent effects, since most studies to date assessing effects of RYGB on intestinal morphology have employed male rodents [12–14].

The aim of this study was to observe the effects of RYGB surgery on intestinal morphology as well as the differential expression of four major gut hormones GIP, GLP-1, GLP-2 and PYY in the BPL compared with the AL in normal rats as well as rats with diet-induced obesity. Our pilot studies warrant detailed follow-up regarding the importance of changes in intestinal morphology and gut hormone populations in the bypassed biliopancreatic and alimentary limb following RYGB.

## Methods

### Animals

Adult female Wistar rats (Envigo Ltd., UK) were housed in the Biomedical and Behavioural Research Unit at Ulster University, Coleraine. At 4 weeks of age, rats were divided into 2 groups (n = 4), that continued on normal diet (ND) standard chow Purina 5008 (4.36 kcal/g, protein 23.6%, fat 6.7%) or were maintained on high-fat diet (HFD, 40% fat, 43% carbohydrate and 17% protein, Special Diet Services, UK). Rats were housed individually in an air-conditioned room at 22 ± 2°C with a 12 h light and 12 h dark cycle. Both standard and high-fat rodent pellet diet and drinking water were available *ad libitum*. Body weight and non-fasting blood glucose were monitored weekly. Only small numbers of rats were used and all animal experiments were carried out in accordance with the UK Animals (Scientific Procedures) Act 1986 and approved by the University of Ulster Animal Welfare and Ethical Review Body (AWERB).

### Surgeries

After 23 weeks on respective diets, rats were further divided into 2 groups resulting in a total of 4 groups which were: ND-sham, ND-RYGB, HFD-sham and HFD-RYGB. Groups of rats underwent sham and RYGB surgeries, as appropriate, at 27 weeks of age. The animals were anesthetised using isoflurane. A single dose of enrofloxacin (7.5 mg/kg—Baytril, Bayer) was administered at induction of anesthesia and buprenorphine (0.02 mg/kg–Animalcare Limited) administered at the end of surgery for postoperative pain. Lacrilube eye ointment was applied to prevent corneal dryness during anesthesia. Following supine positioning, a 2 cm upper midline laparotomy incision was made. The proximal jejunum was divided 10 cm distal to the duodenojejunal flexure to create the BPL and AL. A side-to-side jejunojejunal anastomosis was then formed using a single layer technique with interrupted 6–0 prolene sutures approximately 30 cm from the ileocecal valve. The small bowel of a rat measures about 90cm on average and as such the alimentary limb represents mid and distal jejunum in this model. The lesser curve vessels were carefully dissected, and the gastric cardia divided to create a small gastric pouch (maximum 3 ml volume). The gastric remnant was closed with interrupted 6–0 prolene sutures. An end-to-side gastrojejunal anastomosis was then formed using 6–0 prolene sutures. Warmed, sterile saline (0.9% NaCl, 3.5 ml each side) was administered intraperitoneally before closure. The linea alba was closed with continuous 4–0 Vicryl sutures (Ethicon, Inc.) and the skin closed with subcuticular 4–0 Vicryl sutures. The rats were placed into a clean cage with a warming pad and towel to recover from anesthesia. When fully recovered, they were returned to their home cage. For the sham operation, an upper midline laparotomy incision was made, the small bowel and stomach exposed, and the abdomen was then closed, as described above.

### Post operative care

Post-surgery, the animals were given buprenorphine (0.05 mg/kg, i.p.). From 4 hours post-surgery, water was available *ad libitum*. Rats were housed without bedding for one day to prevent ingestion of bedding material. For 2 days post-surgery, the animals were treated with buprenorphine (0.05 mg/kg, i.p.), as required. On day one and two post-surgery, the rats received 20 ml of oral nutritional supplement liquid diet which was then available *ad libitum* in the afternoon of day 2 (Ensure® Plus, Vanilla, 1.5 kcal/ml, 16.7% protein, 29.5% fat, 53.8% carbohydrate). From days 3–6, the rats were fed wet mash and returned to Ensure if food intake was poor. On days 6–7 post surgery, all rats received standard chow (Purina 5008). Standard chow was provided to all rats as our previous investigations highlight an aversion to HFD, and preference for regular chow, post-surgery in these animals [15]. For 3 weeks post-surgery, all rats were monitored daily for weight loss, food and water intake as well as any signs of infection. Blood samples were collected from the cut tip on the tail vein of conscious rats before surgery and 3 weeks post-surgery. Non-fasting blood glucose was measured directly using a hand-held Ascencia Contour blood glucose meter (Bayer Healthcare, Newbury, Berkshire, UK).

### Tissue processing

Three weeks after surgery, animals were euthanised by lethal inhalation of CO_2_ followed by cervical dislocation. Proximal and distal jejunum was obtained from the sham operated rats to compare with BPL and AL of RYGB rats, respectively. These intestinal tissues were fixed for 48 hours in paraformaldehyde solution (4% w/v in phosphate buffered saline) to preserve cellular architecture by cross-linking proteins. The tissues were then processed in an automated tissue processor which involved dehydrating tissues in 70% to 100% ethanol, followed by xylene immersion to remove ethanol before paraffin embedding. The tissues were then sectioned (5 μm) using a microtome (Shandon Finesse 325, Thermo Scientific) and placed on poly-l-lysine coated slides [16].

### Immunohistochemistry

To assess immunoreactive staining for GIP, GLP-1, GLP-2 and PYY, sections were dewaxed in histoclear for 30 mins before being rehydrated with decreasing concentrations of ethanol. Following this, antigen retrieval and cell permeabilization was achieved using a citrate buffer (pH 6.0) at 96°C for 20 mins followed by a cooling period. The sections were blocked with 2.5% bovine serum albumin (BSA) and then incubated with a primary antibody (Table 1) for the respective peptide overnight. Notably, the GLP-1 antibody employed for our studies will only detect truncated GLP-1 peptides and not related GLP-1 proglucagon gene products such as GLP-1(1–37) [9]. On day 2, the sections were rinsed in phosphate-buffered saline (PBS) twice and incubated with secondary antibody (Alexa Fluor^®^ 488 for green; Table 1) for 1 hour at 37°C. After two further PBS washes, sections were incubated with DAPI for 15 mins at 37°C [16]. Finally, the sections were mounted using antifade and coverslips. Stained sections were viewed at 40x magnification using an Olympus IX51 inverted microscope and photographed using a DP70 digital camera system.

**Table 1:** Target, host and source of primary and secondary antibodies employed for immunofluorescent intestinal histology studies.

**Primary antibodies**			
**Target**	**Host**	**Dilution**	**Source**
PYY	Rabbit	1:500	Abcam, ab22663
GLP-1	Rabbit	1:4	Raised in-house XJIC8
GIP	Rabbit	1:4	RIC34/111J, kindly donated by Professor L Morgan, Guildford, UK
GLP-2	Rabbit	1:100	ThermoFisher Scientific, BS-0208R
**Secondary antibodies**			
**Host and target**	**Reactivity**	**Dilution**	**Fluorescent dilution and source**
Goat IgG	Rabbit	1:500	Alexa Flour 488, Invitrogen, UK

### Image analysis

Image J software was used to assess total BPL and AL area along with their respective villi and crypt area for graphical representation. Only villi that were in the same plane as the slice were analysed. As such, villi length and crypt depth were calculated using the straight-line function. Total number of cells positive for GIP, GLP-1, GLP-2 and PYY, along with their counts in respective villi and crypts, were then counted and expressed as GIP, GLP-1, GLP-2 or PYY positive cells per mm^2^ tissue. This was achieved by employing the closed polygon function in ImageJ to determine the individual area of the ileum and crypt in each image. The area of the crypt was then subtracted from the area of the ileum (crypt + villi) to calculate villi area, with only fully intact villi and crypts analysed. The total number of cells positive for each respective hormone was then divided by the ileum/crypt/villi areas.

### Statistical analysis

GraphPad PRISM software was used to perform statistical analysis. A two-tailed student’s unpaired t-test with 95% confidence interval was used for comparative analysis between groups. Values are expressed as mean ± S.E.M. Difference between groups were significant if p<0.05.

## Results

### AL and BPL morphology after RYGB

**Fig 2:**
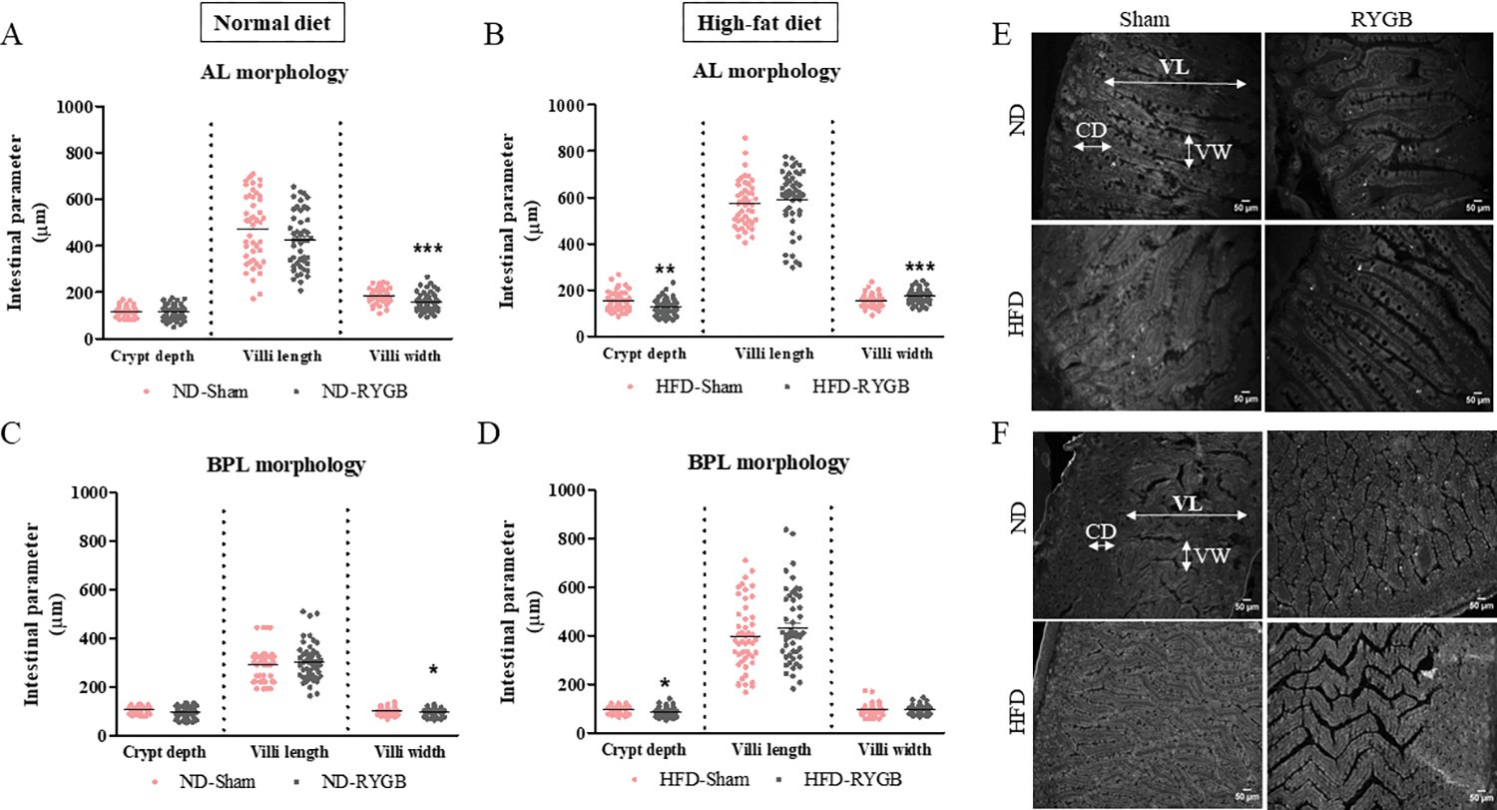
Effect of RYGB on AL and BPL morphology. Graphs show crypt depth, villi length and villi width of (A) Normal diet AL, (B) High-fat diet AL, (C) Normal diet BPL and (D) High-fat diet BPL of female Wistar rats. (E,F) Representative images of AL and BPL where CD-crypt depth, VL-villi length and VW-villi width. Values are mean SEM ± (n = 2); *p<0.05, **p<0.01 and ***p<0.001 compared to respective sham controls.

*In the AL*, RYGB in ND rats significantly (p<0.001) decreased villi width compared to their sham controls (Fig 2A). However, RYGB in HFD rats significantly (p<0.001) increased villi width compared to respective controls (Fig 2B). Additionally, RYGB in HFD rats decreased crypt depth (p<0.01) when compared to their sham control (Fig 2B). *In the BPL*, RYGB in ND rats reduced (p<0.05) villi width (Fig 2C), with no change in crypt depth and villi length. However, RYGB in the HFD group decreased crypt depth (p<0.05) compared to sham rats. Representative images of the AL and BPL are shown in Fig 2E and 2F, respectively.

### GIP-positive cells in the AL and BPL after RYGB

**Fig 3:**
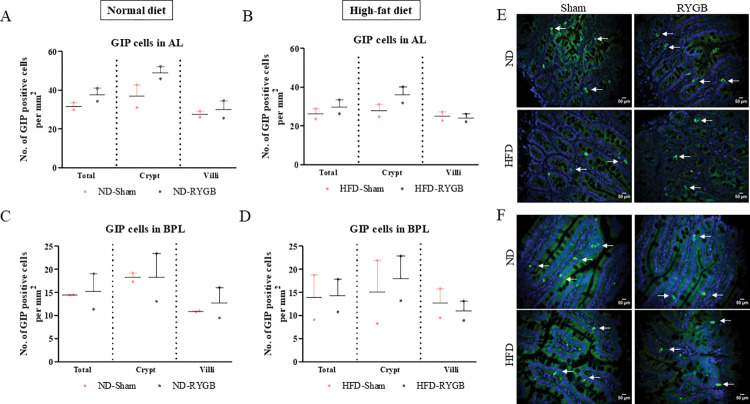
Effect of RYGB on distribution of GIP positive cells. GIP cells per mm^2^ in the total limb, crypt and villi of (A) Normal diet AL, (B) High-fat diet AL, (C) Normal diet BPL and (D) High-fat diet BPL of female Wistar rats. (E,F) Representative images of AL and BPL showing GIP (green) and DAPI (blue). Values are mean SEM ± (n = 2); analyses carried out on ~200 cells (50–60 images) per group.

*In the AL*, numbers of GIP positive cells did not change after RYGB surgery in both ND and HFD rats (Fig 3A and 3B; representative images in Fig 3E). *In the BPL*, RYGB did not significantly affect the number of GIP positive cells per mm^2^ of total limb, crypt or villi in both ND and HFD rats (Fig 3C and 3D; representative images in Fig 3F).

### GLP-1-positive cells in the AL and BPL after RYGB

**Fig 4:**
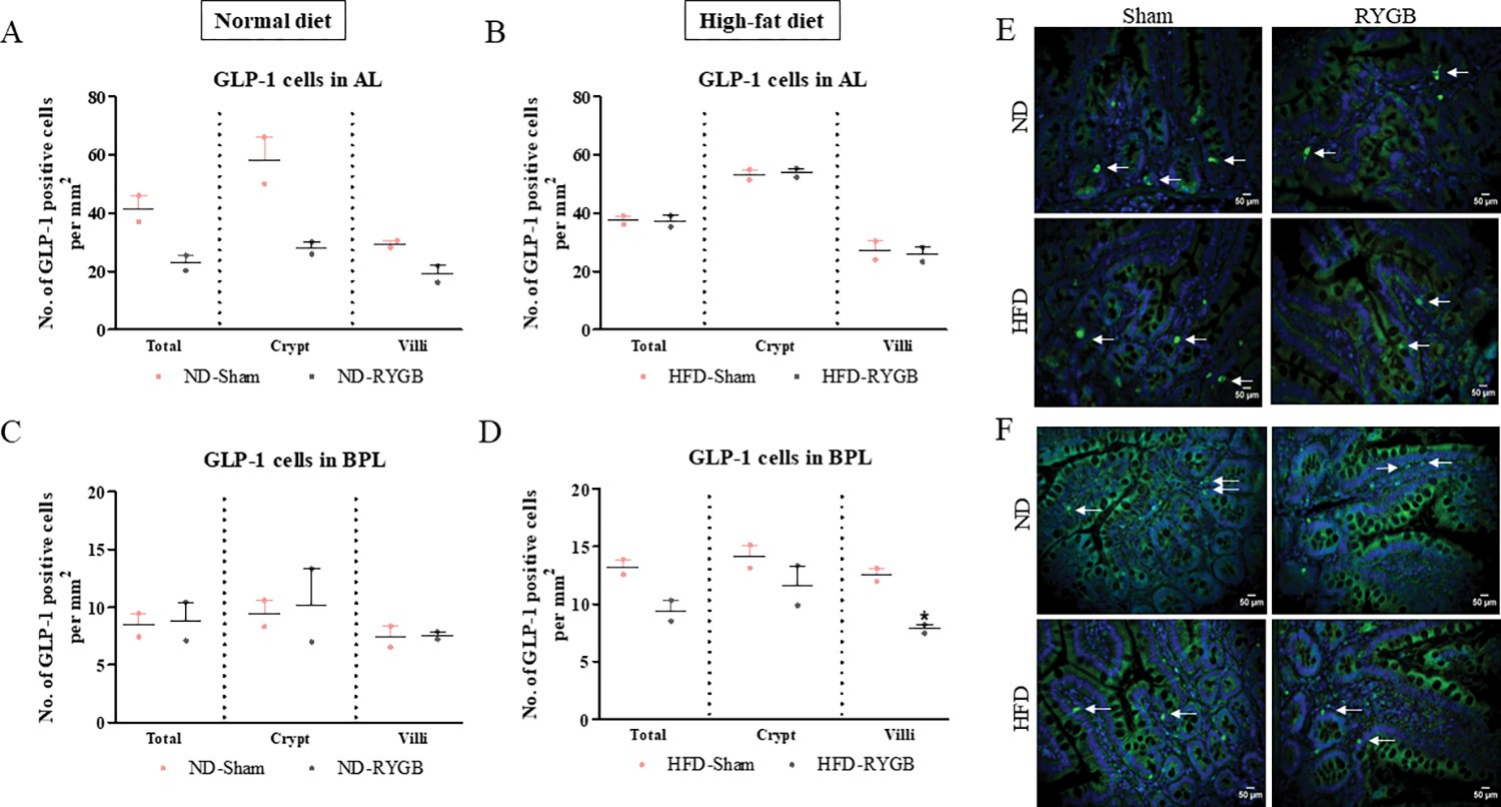
Effect of RYGB on distribution of GLP-1 positive cells. GLP-1 cells per mm^2^ in the total limb, crypt and villi of (A) Normal diet AL, (B) High-fat diet AL, (C) Normal diet BPL and (D) High-fat diet BPL of female Wistar rats. (E,F) Representative images of AL and BPL showing GLP-1 (green) and DAPI (blue). Values are mean SEM ± (n = 2); analyses carried out on ~200 cells (50–60 images) per group. *p<0.05 compared to respective sham controls.

*In the AL*, numbers of GLP-1 positive cells in the RYGB crypt and villi did not change in ND and HFD rats compared to their respective sham controls (Fig 4A and 4B; representative images in Fig 4E). *In the BPL*, RYGB did not significantly affect the number of GLP-1 positive cells per mm^2^ in the total limb and crypt, but did decrease (p<0.05) villi cell numbers in HFD, but not ND, rats (Fig 4C and 4D; representative images in Fig 4F).

### GLP-2-positive cells in the AL and BPL after RYGB

**Fig 5:**
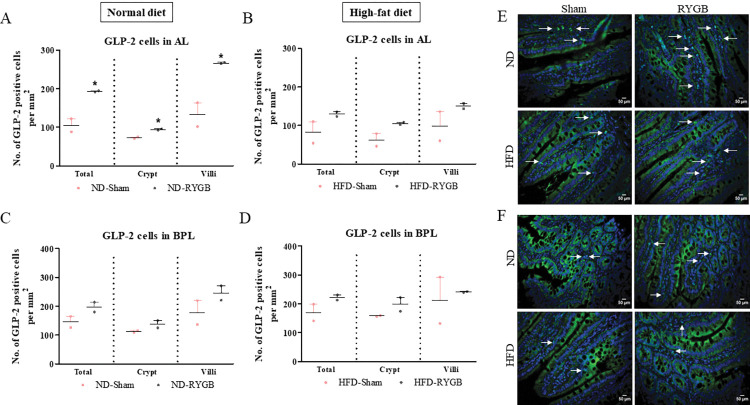
Effect of RYGB on distribution of GLP-2 positive cells. GLP-2 positive cells per mm^2^ in the total limb, crypt and villi of (A) Normal diet AL, (B) High-fat diet AL, (C) Normal diet BPL and (D) High-fat diet BPL of female Wistar rats. (E,F) Representative images of AL and BPL showing GLP-2 (green) and DAPI (blue). Values are mean SEM ± (n = 2); analyses carried out on ~200 cells (50–60 images) per group. *p<0.05 compared to respective sham controls.

*In the AL*, RYGB in ND rats induced a significant (p<0.05) increase in the number of GLP-2 positive cells in the crypt and villi, as well as total AL, when compared to their sham controls (Fig 5A). However, there was no change in the number of GLP-2 positive cells in the HFD group after RYGB (Fig 5B; representative images in Fig 5E). *In the BPL*, there was no significant difference in the number of GLP-2 positive cells per mm^2^ between the RYGB and sham groups in both ND and HFD animals (Fig 5C and 5D; representative images in Fig 5F).

### PYY-positive cells in the AL and BPL after RYGB

**Fig 6:**
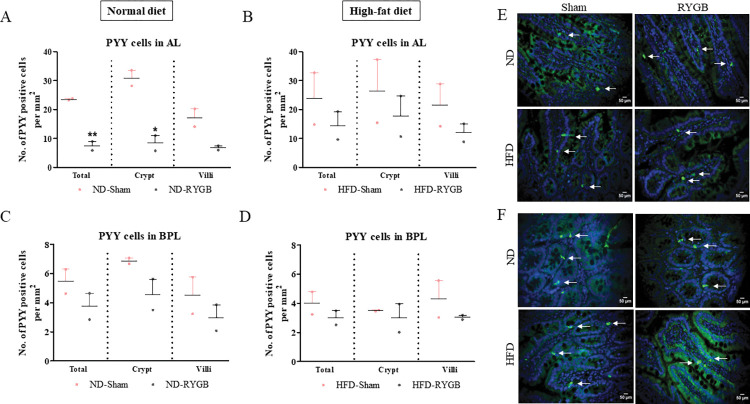
Effect of RYGB on distribution of PYY positive cells. PYY positive cells per mm^2^ in the total limb, crypt and villi of (A) Normal diet AL, (B) High-fat diet AL, (C) Normal diet BPL and (D) High-fat diet BPL of female Wistar rats. (E,F) Representative images of AL and BPL showing PYY (green) and DAPI (blue). Values are mean SEM ± (n = 2); analyses carried out on ~200 cells (50–60 images) per group. *p<0.05 and **p<0.01 compared to respective sham controls.

*In the AL*, RYGB surgery in ND rats caused a significant (p<0.05 to p<0.01) decrease in the number of PYY positive cells per mm^2^ of total limb and crypt but not villi, when compared to their sham controls (Fig 6A). RYGB did not change the distribution of PYY positive cells in total limb, crypt and villi of HFD rats (Fig 6B; representative images in Fig 6E). *In the BPL*, RYGB did not significantly affect the number of PYY positive cells per mm^2^ in the crypt and villi of both ND and HFD rats (Fig 6C and 6D; representative images in Fig 6F).

### Body weight and non-fasting blood glucose after RYGB

**Fig 7:**
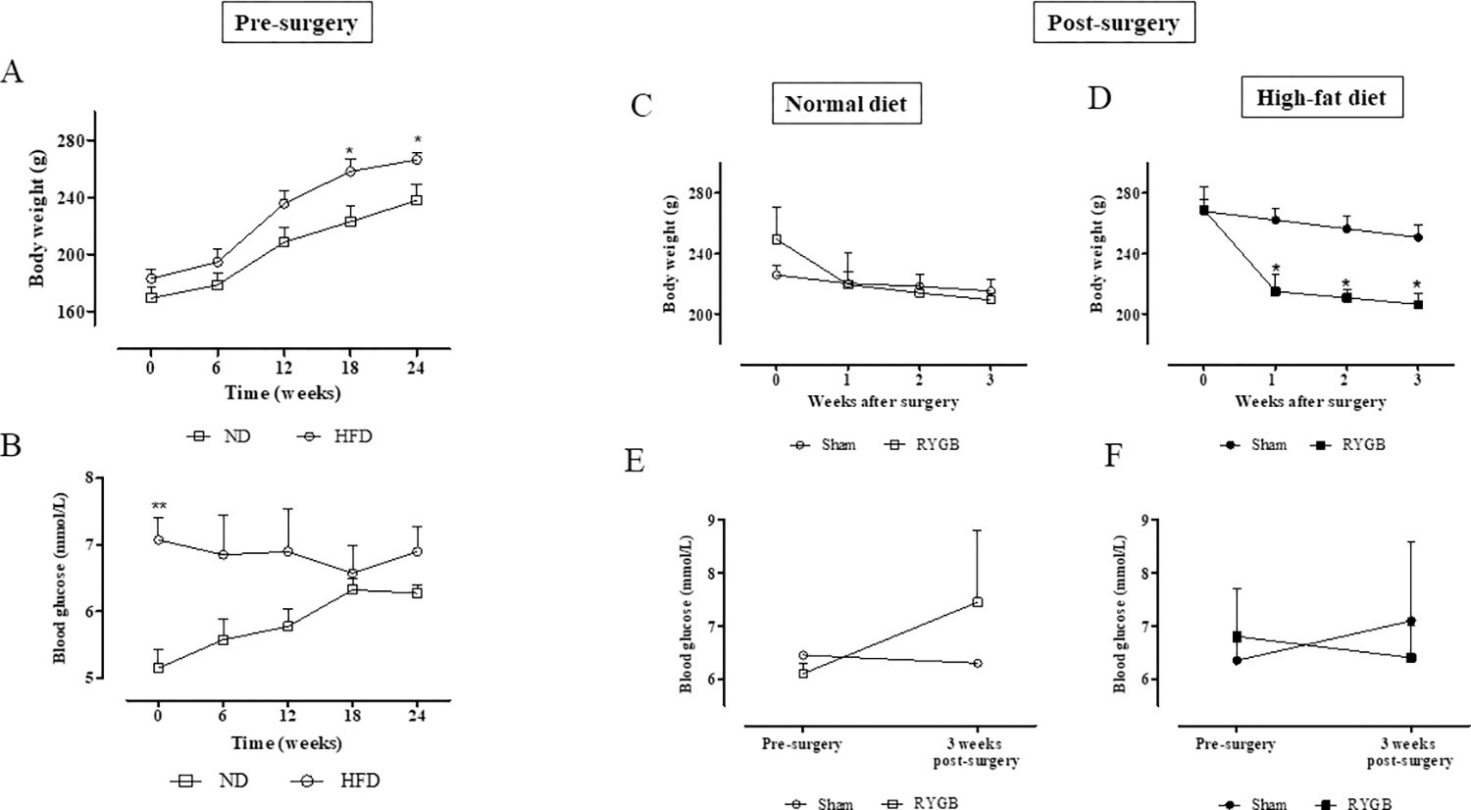
Effect of HFD and RYGB on body weight and non-fasting blood glucose. Body weight (A) and non-fasting blood glucose (B) of ND and HFD rats from week 0 to 24 before RYGB surgery. Post-surgery body weights of sham and RYGB rats fed ND (C) and HFD (D) as well as related non-fasting blood glucose levels (E,F). Values are mean SEM ± (n = 2). *p<0.05 and **p<0.01 compared to ND and respective sham controls.

Prior to surgery, body weights of HFD rats were significantly (p<0.05) increased when compared to ND rats (Fig 7A). Non-fasting blood glucose was marginally elevated at the commencement of the HFD regimen when compared to control rats (7.0 ± 0.6 *vs*. 5.2 ± 0.5 mmol/l; p<0.01), but not different pre-surgery (Fig 7B). While body weights of all rats undergoing sham surgery remained relatively stable, there was an approximate 10% decrease of body weight in ND rats following RYGB (Fig 7C), with a significant (p<0.05) 20% body weight decrease in the HFD-RYGB group of rats (Fig 7D). No significant differences in non-fasting blood glucose were observed in any of the groups of rats after surgery (Fig 7E and 7F).

## Discussion

In this small pilot study, RYGB was associated with relatively minor changes in intestinal morphology in rats, which were nevertheless greater in the AL than in the bypassed BPL. We observed decreased crypt depth and increased villi width in the AL of HFD-RYGB rats, with no change in villi length, in good agreement with others [12,17]. This may indicate villi hypertrophy, a hallmark of mucosal adaptation, in response to increased nutrient stimulation [13]. Thus, our observations are consistent with intestinal hyperplasia in the alimentary limb of these rats, which has been described before [13,18]. However, in ND-RYGB rats, AL villi width was decreased, suggesting previous nutrient composition, rather than levels of intestinal cell stimulation *per se*, play a key role in these changes of intestinal morphology. In the BPL, one notable observation in terms of intestinal morphology was a small but significant decrease in villi width following RYGB in ND rats, which was not apparent under conditions of previously sustained high fat feeding. Interestingly, BPL crypt depth was decreased only in HFD rats after RYGB. These changes may simply reflect re-direction of digesta away from the BPL post-surgery.

Whilst intestinal morphology was not dramatically altered by bypass surgery, it seemed reasonable to further investigate gut hormone populations in the AL and BPL of ND- and HFD-RYGB rats. GLP-1 has long been advocated as the chief hormonal protagonist for RYGB induced metabolic benefits [19], originating largely from L-cells located in the ileum [20]. As such, GLP-1 delays gastric emptying, promotes satiety together with weight loss and improves glucose tolerance through stimulation of glucose-dependent insulin secretion [21]. Nevertheless, we observed no changes in GLP-1 cell density post-surgery in the AL of both experimental groups. This is in broad agreement with others [13,17], and suggests that increased postprandial GLP-1 levels following RYGB are linked to expediated delivery of nutrients and bile acids to the AL to stimulate secretion [22]. Consistent with this view, RYGB did not alter the intestinal population of GIP positive cells. Whilst others have demonstrated decreased [23] or increased [17,24] GIP intestinal cell density post-surgery, our findings argue against the idea that diabetes amelioration after RYGB is due to loss of EECs secreting the hormone GIP [25] or other hormones from the BPL [26].

Also secreted from the L-cells is the intestinotrophic hormone GLP-2 which is derived from alternative processing of the proglucagon gene. This hormone is not entirely devoid of metabolic effects [27], but is believed to play a major role in the regulation of intestinal cell proliferation [28]. Indeed, the potential of GLP-2 to stimulate intestinal regeneration has been well studied in rats and mice [29,30]. As expected, we observed prominent increases in GLP-2 cell density in the AL of ND-RYGB rats in good agreement with previous human [8,11,31] and rodent [32] investigations. Our work implies that RYGB induces a greater proportion of L-cells to process proglucagon towards GLP-2 rather than to GLP-1 synthesis, although it is notable that numbers of GLP-1 expressing cells were maintained. PYY is another peptide hormone synthesised by enteroendocrine L-cells of the AL. Although PYY has no structural association to GLP-1, the major circulating metabolite, namely PYY (3–36), also induces satiety and weight loss through modulation of NPY2 receptors [33,34]. Interestingly, numbers of PYY expressing cells were decreased in the AL after RYGB in ND rats, a mirror image to the increase of GLP-2. Thus, complementary changes of GLP-2 and PYY expression within L-cells of the AL may provide important clues towards some of the metabolic benefits following RYGB. There is a well described differential pattern of hormone expression along the length of the intestine [35] which may have an impact on the number of hormone positive cells detected in our study. For example, the proximal intestine is considered to harbour more GIP positive cells whereas PYY cells are more abundant in distal regions of the gut [36].

The BPL is devoid of nutrient stimulation following RYGB, and as a result the impact of surgery on morphology and gut hormone populations within this section of the intestine has been somewhat overlooked. In our setting, only negligeable intestinal atrophy was detected in the BPL of ND-RYGB rats, and the solitary alteration of gut hormone population in this bypassed section of gut was a reduction in GLP-1 expression in the villus, but not crypts, in HFD-RYGB rats. Thus, GLP-1 positive cells are more predominant in intestinal crypts rather than villi [37], and increased GLP-1 cell numbers in the villi may simply reflect adaptation to high fat feeding prior to surgery, that is fully reversed by RYGB. In this respect, EECs do possess an ability to switch hormone expression along the crypt-to-villus length [38], and high fat feeding has been demonstrated to reduce expression of GLP-1 specific genes and impair secretory function in rodents [39]. EECs also have a turnover rate of approximately 3–5 days [40], that needs to be considered in the context of the current observations. Nonetheless, when taken together our data clearly indicate that isolation from the food stream does not have an appreciable adverse effect on gross morphology or gut hormone population of the BPL, which maintains a healthy appearance and presumably function. Enhanced production and local effects of GLP-2 may partly explain intact intestinal morphology of the BPL despite of loss of enteral stimulation, although there was only a tendency for increased numbers of GLP-2 positive cells in the BPL post-surgery, which was more prominent the AL.

In conclusion, our findings alongside recently published literature, suggest that early adaptation of EECs in the gut after RYGB surgery accompany the improved metabolic state. Although the pilot nature and relatively small sample size employed for the current study means it is difficult to make emphatic claims in this regard, the work does represent a firm initial standpoint for elucidation of such mechanisms. Notably, the morphology and EEC populations of the BPL are not appreciably affected by the bypass, arguing against the idea that metabolic benefits of RYGB are due to exclusion of factor derived from this intestinal region. However, despite the small number of rats studied, villi area in the AL was clearly augmented together with reciprocal changes of GLP-2 and PYY expression post-surgery. Such changes merit further exploration in terms of their direct impact to the well characterised metabolic benefits of RYGB surgery.
